# ASC-H in Pap test- definitive categorization of cytomorphological spectrum

**DOI:** 10.1186/1742-6413-3-14

**Published:** 2006-05-10

**Authors:** Mamatha Chivukula, Vinod B Shidham

**Affiliations:** 1 Department of Pathology, Magee-Womens Hospital of University of Pittsburgh Medical Center, Pittsburgh, PA, USA; 2 Department of Pathology, Medical College of Wisconsin, Milwaukee, WI, USA

## Abstract

**Objective:**

The American Society for Colposcopy and Cervical Pathology (ASCCP) guidelines for management of ASC-H is colposcopic examination followed by biopsy. HPV testing (HPVT) is recommended after a negative biopsy result. More definitive interpretation of ASC-H could prevent discomfort and minimize the cost. The purpose of this study was to evaluate association of various cytomorphological patterns of ASC-H with various clinical scenarios.

**Methods:**

We reviewed SurePath™ (TriPath Imaging, Inc. Burlington, NC, USA) cervical smears interpreted as ASC-H in 161 women (mean age, 37 {15 to 78} years), over 24 months (2002 to 2003). HPVT (Digene, Hybrid Capture^® ^II HPV test, Digene Corporation, Gaithersburg, MD, USA) was performed in 20% of cases (33/161) and biopsy results were available in 54 cases (19 with and 35 without HPVT).

**Results:**

HPVT was *positive *in 64% (21/33) cases, and negative in 36% (12/33) cases. In the follow-up biopsies of 71% (15/21) of cases with positive HPVT, 27% showed HPV changes or CIN1, 27% showed CIN2-3, and 46% were negative for epithelial abnormality. Follow-up biopsies from cases with negative HPVT (33%, 4/12 cases), 8% showed CIN1 and 25% were negative for any epithelial abnormality. Six cytomorphological patterns of ASC-H correlated with different clinical categories in relation to HPVT and biopsy results. 35% (19 out of 54 ASC-H cases in which biopsy results were available) could be interpreted definitively as HSIL by cytopathology, 11% (6/54) cases as LSIL with cyanophilic atypical parakeratotic pattern, and 31% (17/54) cases as reactive, with HPV status. The interpretation had to be continued as ASC-H in 22% (12/54) cases.

**Conclusion:**

ASC-H demonstrated a spectrum of cytomorphological patterns. Some of these patterns in liquid-based cervical smears may be more specifically interpreted as LSIL, HSIL, or benign if HPV status is known.

## Background

'Atypical squamous cells- cannot exclude HSIL' (ASC-H) was recognized in 2001 Bethesda System [1,2] and [3]. The previous category of ASCUS was replaced by (a) 'ASC of undetermined significance' (ASC-US) and (b) ASC-H [1,2]. This new category includes cases with atypical squamous cells that exhibit some equivocal features suggestive of but not sufficient to call HSIL. The cytomorphological criteria that can be applied for interpretation of this category are not well defined in the literature in general with most of the methods including liquid based cytology (LBC) techniques such as SurePath™ (Tri Path Imaging, Inc, Burlington, NC, USA) and many newly described alternatives [4].

Although relatively an uncommon interpretation, they are associated clinically with significant lesions in the cervical biopsies [6,7] and also anal canal lesions [16,17]. The guidelines of American Society for Colposcopy and Cervical Pathology [11] for management of ASC-H in cervical smears are colposcopic examination followed by biopsy confirmation. If a lesion is not identified on colposcopic examination, the cervical smear is reviewed again followed by a repeat cervical smear at 6 or 12 months or HPV-DNA testing (HPVT) at 12 months.

The purpose of our study is to evaluate various cytomorphological patterns observed in cervical smears previously interpreted and reported as ASC-H in SurePath™ LBC. Different cytomorphological patterns were correlated with reference to results of HPVT (Digene, Hybrid capture ^® ^II HPV test, Digene Corporation, Gaithersburg, Maryland, USA) and biopsy findings. We analyzed if HPV status through HPVT results could be used as an ancillary test to help categorize different cytomorphological patterns more specifically after initial ASC-H interpretation.

## Materials and methods

A total of 161 cases with cytological interpretation of ASC-H in SurePath™ liquid-based cervical cytology smears over a period of 24 months (2002–2003) were studied. The mean age of women in the study was 37 (range 15–78) years. HPVT was performed on remaining specimens in 33 (21%) cases. The respective cervical biopsies obtained synchronously or within 3 months of cervical smear collection of cervical smears were identified, reviewed and correlated in 54 (34%) cases. Our cytology laboratory processes pap tests from our own hospital as well as outside clinics. Since the outside clinics did not send their biopsies to our lab, we could not correlate biopsy results in all cases.

Based on the presence or absence of CIN 2, CIN 3 or above in the biopsies and the status of HPVT results, all the cases were grouped into 6 clinicopathological categories [Table 1]. A) Biopsy positive for CIN 2, CIN 3 or above (BPHSIL) HPVT positive B) biopsy negative for CIN 2, CIN 3 or above (BNHSIL), HPVT positive C) biopsy positive for CIN 2, CIN 3 or above (BPHSIL), HPVT negative D) biopsy negative for CIN 2, CIN 3 or above (BNHSIL), HPVT negative E) biopsy positive for CIN 2, CIN 3 or above (BPHSIL) HPVT not done F) biopsy negative for CIN 2, CIN 3 or above (BNHSIL), HPVT not done.

**Table 1:** Clinicopathological categories.

	CATEGORY	# OF CASES
**A**	HPV test and biopsy both positive	**11**
**B**	HPV test positive and biopsy negative for CIN II-CIN III	**4**
**C**	HPV test negative, biopsy positive	**1**
**D**	HPV test negative and biopsy negative for CIN II-CIN III	**3**
**E**	HPV test not done and biopsy positive	**18**
**F**	HPV test not done and biopsy negative for CIN II-CIN III	**17**

The cytomorphological features in all cervical smears interpreted as ASC-H were evaluated retrospectively in conjunction with the results of cervical biopsies and HPVT results. A trend in association of various cytomorphological patterns was observed in relation to above-mentioned six clinicopathological categories [Table 2 &Additional file 1].

**Table 2:** Cytomorphological Patterns associated with different clinicopathological categories.

		**Clinicopathological categories**					
		**A**	**B**	**C**	**D**	**E**	**F**
Cytomorphological Patterns		**H- P** **B- P**	**H- P** **B- N**	**H- N** **B- P**	**H- N** **B- N**	**H- ND** **B- P**	**H- ND** **B- N**
**Reactive**	**1**. *MGH-like*	0	0	0	2	0	8
	**2. ***Repair like*	0	0	0	0	0	4
	**3. ***Atrophy like*	0	0	0	1	0	2
**Indeterminate**	**4. ***ASC-H: NOS*	3	1	1	0	4	3
**LSIL**	**5. ***Cyanophilic atypical parakeratosis*	3	1	0	0	0	0
**HSIL**	**6A ***HSIL- syncytial*	2	1	0	0	2	0
	**6B ***HSIL- Single-cell*	2	0	0	0	12	0
	**Total**	**11**	**4**	**1**	**3**	**18**	**17**

H- HPV testing, B- Biopsy, P- positive, N-Negative, ND- not done

## Results

HPVT results were positive in 64% (21/33) cases. Out of these 21 cases with positive HPVT, biopsies were available in 15 cases which showed HPV changes or CIN-1 in 27% (4/15), CIN 2–3 in 27% (4/15), and negative for dysplasia in 47% (3/15) cases. HPVT was negative in 36% (12/33) cases. Out of these 12 HPVT negative cases, biopsies were available in 4 cases which showed CIN-1 in 25 % (1/4) and negative for dysplasia in 75% (3/4).

Out of 128 cases without HPVT, cervical biopsies were available in 35 cases (27.3%). The biopsies in these cases showed CIN-1 in 27% (13/35), CIN 2–3 in 11% (4/35), and negative for dysplasia in 51 % (18/35) cases.

Six cytomorphological patterns [Additional file 1] were correlated with 6 different clinico-pathological categories A through F [Table 1] and a trend in their association was observed [Table 2]. Reactive cytomorphological patterns 1, 2, &3 were associated with categories D & F with negative biopsy results. Dysplastic patterns 5, 6, &7 were associated with categories A & E with positive biopsy (for HPV and/or dysplasia).

**Figure 1:**
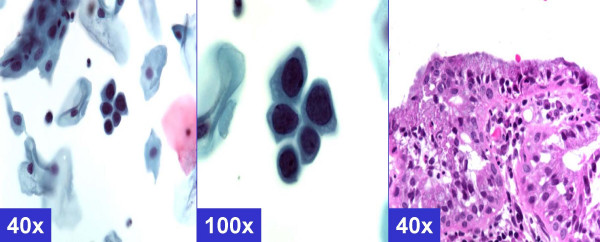
*MGH-like pattern *(ASC-H, favor reactive) Groups of metaplastic cells arranged in checkerboard like pattern. The dark nuclei may show nucleoli (arrows). (A & b- Cervical smear [Papanicolaou stained SurePath™ Preparation], c- Cervical biopsy [Hematoxylin-eosin stained section]).

**Figure 2:**
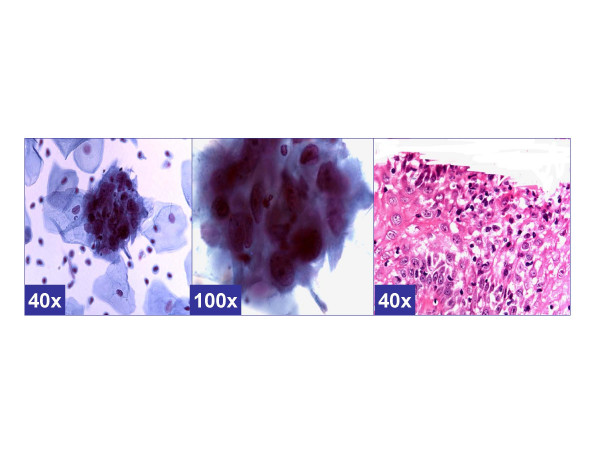
*Repair-like pattern *(ASC-H, favor repair). Cohesive groups of cells with ill-defined school of fish pattern with relatively polarized cells with pointed ends (arrow head) show relatively low N/C ratio. The nuclei show nucleoli (arrows). (a & b- Cervical smear [Papanicolaou stained SurePath™ Preparation], c- Cervical biopsy [Hematoxylin-eosin stained section]).

**Figure 3:**
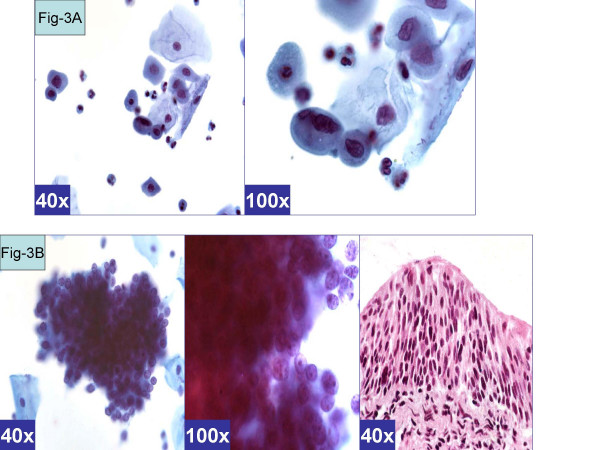
*Atrophy-like pattern *(ASC-H, favor atrophy). **A**. Single cell pattern. Isolated cells with hyperchromatic atypical nuclei with smudgy chromatin). (a & b- Cervical smear [Papanicolaou stained SurePath™ Preparation]). **B**. HCG of parabasal cells. Cohesive hyperchromatic crowded groups of small parabasal cells with high N/C ratio. The nuclei are relatively small and show nucleoli (arrow). (a & b- Cervical smear [Papanicolaou stained SurePath™ Preparation], c- Cervical biopsy [Hematoxylin-eosin stained section]).

The *reactive *cytomorphological patterns [Table 2 &Additional file 1] included *MGH-like*, *repair- like*, or *atrophy-like *patterns. *MGH-like pattern *[Figure-1] showed groups of immature squamous metaplastic cells arranged in a checkerboard pattern [5]. The cells had dark nuclei with open chromatin. Nucleoli may be present. Normoblast-like apoptosis with apoptotic fragments of karyorrhectic nucleus may be observed in area corresponding with the nucleus in some metaplastic cells [5]. The *repair-like pattern *[Figure-2] showed cohesive clusters of cells with prominent nucleoli and streaming school of fish like pattern. The *atrophy-like pattern *[Figure-3] showed either *single-cell pattern *or *hyperchromatic crowded groups (HCG) of para-basal cells*. In the *single cell pattern *[Figure-3A], the individual cells had abundant blue cytoplasm, open chromatin with or without nucleoli. On the other hand *groups of parabasal cells as HCG *[Figures-3B] showed small dark nuclei and variable cytoplasm usually with low N/C ratio. The chromatin was dark but not clumped.

*ASC-H: NOS pattern *did not show any specific cytological features, but showed atypical squamous cells with high N/C ratio and hyperchromatic nuclei with coarse chromatin.

Six cases with cyanophilic *atypical parakeratosis *(CAPK) pattern [Table 2, pattern 5, Figure-5] showed cohesive HCG of immature, relatively small, metaplastic cells with high nuclear to cytoplasmic ratio and relatively cyanophilic staining pattern. Some showed koilocytosis. The cell margins in the group were relatively well seen and were angulated as compared to ill defined curved round cell margins at the periphery in high-grade cells with syncytial pattern. The cervical biopsies showed HPV cytopathic effect with or without mild dysplasia in 3 of these 6 cases.

**Figure 4:**
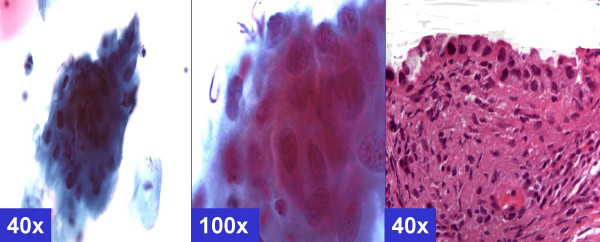
*ASC-H: NOS*. Cohesive groups of atypical cells with mostly ill-defined cell borders. The nuclei vary in size with coarse chromatin; however, the nuclear details in most are relatively smudgy (arrows). (a & b- Cervical smear [Papanicolaou stained SurePath™ Preparation], c- Cervical biopsy [Hematoxylin-eosin stained section]).

**Figure 5:**
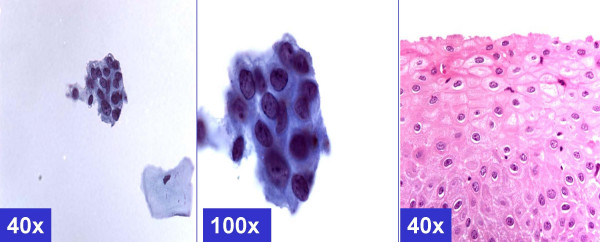
*Cyanophilic Atypical parakeratosis *(ASC-H, favor HPV). Cohesive groups of hyperchromatic cyanophilic cells with ill-defined cell borders, which are straight with angulations better seen at periphery (arrow). N/C ratio is higher. Chromatin is smudgy. Some cells may show koilocytic space around nuclei. (a & b- Cervical smear [Papanicolaou stained SurePath™ Preparation], c- Cervical biopsy [Hematoxylin-eosin stained section]).

**Figure 6:**
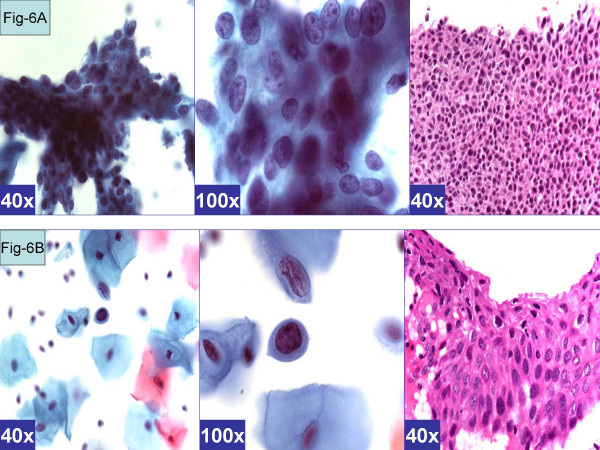
*HSIL pattern *(ASC-H, favor HSIL). **A. **Syncytial pattern. Hyperchromatic crowded groups of cells without distinct cell borders. The hyperchromatic nuclei vary in size and show coarsely granular chromatin (arrows). (a & b- Cervical smear [Papanicolaou stained SurePath™ Preparation], c- Cervical biopsy [Hematoxylin-eosin stained section]).**B. **Singly scattered (so called 'litigation') cells. Scattered, isolated, atypical cells show high N/C ratio. The nuclei have coarse chromatin without nucleoli (arrows). (a & b- Cervical smear [Papanicolaou stained SurePath™ Preparation], c- Cervical biopsy [Hematoxylin-eosin stained section]).

The *HSIL like pattern *[Figure-6] showed either *single-cell pattern *or *syncytial pattern*. The *ASC-H with syncytial pattern *[Figure-6A] showed vague syncytial groups of dysplastic cells, which may show focal single cell apoptosis with randomly scattered apoptotic bodies in the apoptotic cells [5]. The nuclear features included hyperchromasia, coarse chromatin, and absence of nucleoli.

*ASC-H with HSIL-like pattern of singly scattered cells *[Figure-6B] showed isolated single cells similar to 'litigation cells'. These cells with high N/C ratio showed hyperchromatic nuclei without nucleoli.

## Discussion

ASC-H is a new category included in the Bethesda Classification 2001 under the epithelial cell abnormalities [2]. It includes approximately 5–10% of previous ASC-US cases and mimics of HSIL [1,6]. Several previous studies have shown that the positive predictive value for detection of high-grade dysplasia in this group is significantly higher than with ASC-US group [9,12,13].

*Selvaggi et al *reported 68% of high-grade dysplasia in follow-up cervical biopsies in cases with ASC-H [6]. *Ali et al *reported 48% CIN-1 and 51% CIN 2–3 out of 257 cervical smears with ASC-H, of which 72 had follow-up biopsies [9]. In another study *Raab et al *reported 26% of high-grade dysplasia detected in the follow-up biopsies of all the cervical smears with ASC-H [12]. The variation in cytohisto correlation pattern reported by different studies may be due to many factors including level of threshold applied to interpret the cytomorphological features as ASC-H or HSIL in a particular lab. These studies further highlight the challenges due to lack of well-defined cytomorphological criteria for ASC-H interpretation.

This suggests a need for well defined specific cytomorphological criteria to help categorize such cervical smears as ASC-H. A very few studies in literature describe the cytomorphological features associated with ASC-H interpretation. Our attempt in this paper is to study the cytomorphology of all the smears interpreted as ASC-H to get a better understanding of the various patterns associated with it. Shidham et al have reported that metaplastic pattern associated with microglandular hyperplasia may lead to ASC-H interpretation [5]. In a study by Selvaggi et al, the most commonly reported findings as ASC-H were atypical squamous metaplastic cells and disorganized HCG [6].

In our study, review of various cytomorphological features [Additional file 1] in LBC smears with reference to different clinico-pathological categories [Table 1] revealed a pattern of association [Table 2].

The cytomorphological features observed in category D (with BNHSIL and negative HPVT) and category F (with BNHSIL) showed benign reactive patterns such *MGH-like pattern *demonstrating dyshesive cells in checker-board groups [Additional file 1, pattern 1, Figure 1];*repair-like pattern *with streaming groups of cells [Additional file 1, pattern 2, Figure 2]; and *atrophy-like pattern *with hyperchromatic crowded groups of parabasal cells [Additional file 1, pattern 3 A&B, Figures A&B]. While none in this clinical category showed cyanophilic atypical parakeratosis (pattern 5), HSIL synticial (pattern 6), HSIL single-cell patterns [Table 2].

On the other hand syncytial groups of cells with high N/C ratio with focal individual cell apoptosis could be interpreted as high-grade intraepithelial neoplasia [Additional file 1, pattern 6B, Figure 6B]. Rare cases showed some of these cells as rare single cells [Additional file 1, pattern 6A, Figure 6A]. This pattern was observed in association with cases with CIN2–CIN3 or above in biopsy, i.e. categories A (BPHSIL and HPVT positive) and E (BPHSIL, HPVT negative).

The term 'atypical parakeratosis' (APK) was described in original Bethesda classification under the ASCUS/SIL category as three-dimensional clusters that demonstrated the cellular pleomorphism with caudate or elongated shapes with increased N/C ratio or hyperchromasia. In conventional smears, these groups usually show significant orangeophilia and usually have a differential diagnosis with LSIL. We observed similar groups in SurePath LBC initially interpreted as ASC-H. However, these groups were usually cyanophilic with rare or none orangeophilic cells. In general, the cells in SurePath LBC preparations shrink and appear smaller than conventional smears and so the cells in these groups also appear small. These cyanophilic groups with relatively small cells overlap in morphology with HSIL. They were interpreted as ASC-H and showed association with HPV or CIN1 in biopsy with positive HPVT. This pattern may be categorized as *ASC-H, favor HPV*. Usual atypical parakeratosis encompasses low-grade differential diagnosis; however, in SurePath LBC some of these groups overlapped in morphology with HSIL, leading to ASC-H interpretation. As most of these groups were cyanophilic, a terminology 'cyanophilic atypical parakeratosis' (CAPK) [Additional file 1, pattern 5, Figure 5], is used in this article to differentiate them from 'atypical parakeratosis in conventional smears with LSIL differential diagnosis.

### Role of HPV-DNA testing in ASC-H cases

According to ASCCP guidelines [8] HPVT for high-risk (oncogenic) HPV is recommended in cases with ASC-US. On the other hand the recommended management of ASC-H is colposcopy. Studies have reported association with high-risk HPV in 37.5% [13] to 71% [14] of ASC-H cases. The ASCUS-LSIL Triage Study (ALTS) observed an association of ASC-H with positive HPVT and high-grade lesion (biopsy showing CIN-2 or above) in 30–40% cases, which is higher than with usual ASCUS (10–15% high grade) [1]. This study showed that positive HPVT correlated with ASC-H patterns associated with positive biopsy results (Table 2). Thus in future HPVT may be applied in similar fashion to that of ASCUS and it may be ordered reflexly in all ASC-H cases.

Other important aspect of the finding of this study is that HPVT results may be applied to modify the preliminary ASC-H interpretation, although this is not usual recommended role of HPVT as compared to ASCUS. In this study, reactive and atrophic ASC-H patterns were associated with negative HPVT. In contrast, ASC-H patterns with LSIL-HSIL on biopsy showed higher chance of positive HPVT (Table 2). In future, in-situ hybridization may be performed to identify the viral genome in the abnormal ASC-H cells. This may be performed [2] directly on parallel smears or directly on the de-stained smears showing ASC-H at initial interpretation to confirm the HPV status of the abnormal cells under question. Recently reported immuno marker p16^INK4 ^may also be incorporated for definitive interpretation of ASC-H with relevant precautions by avoiding pitfall of interpreting non-nuclear immunostaining as significant [18,19].

In summary, this study identified a cytomorphologic spectrum (Additional file 1, Figures 1 through 6) associated with ASC-H interpretations in SurePath LBC preparations in our laboratory, which demonstrated a pattern with biopsy and HPVT results (Table 2).

Six cytomorphological patterns in association with 6 clinico-pathological categories endorses that ASC-H is not a homogeneous category. A blinded study in future may refine the observations of this study. The patterns ranged from reactive to indeterminate (NOS) to dysplasia [Table 2, Figures 1 through 6]. If HPVT is negative and ASC-H pattern resembles one of the reactive patterns [Table 2], the findings may be interpreted definitively as reactive with a note-recommending follow-up. Similarly, if HPVT is positive for high risk HPV and ASC-H pattern is more close to that of dysplastic type, the case could be interpreted as HSIL. HPVT positivity with atypical parakeratosis pattern may be interpreted definitively as LSIL or ASC-H favor LSIL. Any other combination where the results of HPVT and the cytomorphological features are not in congruency may be continued as ASC-H- NOS.

## Abbreviations

ASC, Atypical squamous cells; APK, Atypical parakeratosis, ASC-H, Atypical squamous cells- cannot exclude high-grade squamous intraepithelial lesion; ASC-US, Atypical squamous cells of undetermined significance; BNHSIL, biopsy negative for CIN 2, CIN 3 or above; BPHSIL, biopsy positive for CIN 2, CIN 3 or above; CAPK, Cyanophilic atypical parakeratosis; HCG, hyperchromatic crowded groups; HPVT, HPV-DNA testing; HSIL, high grade squamous intraepithelial lesion; LBC, liquid-base cytology; LSIL, low grade squamous intraepithelial lesion. BPHSIL, biopsy positive for CIN 2, CIN 3 or above, BNHSIL, biopsy negative for CIN 2, CIN 3 or above.

## Authors' contributions

1. (MC) Cytopathology fellow, collected all the data, participated in cytological evaluation, and drafting of manuscript.

2. (VS) Mentor, conceptual organization, cytological-histological evaluation, and manuscript writing.

## Supplementary Material

Additional File 1 Schematic representation of various cytomorphological patterns observed with ASC-H interpretation. Click here for file
