# Supplementary material for: ASC-H in Pap test- definitive categorization of cytomorphological spectrum
Source: Cytojournal. 2006 May 10;3:14. doi: 10.1186/1742-6413-3-14 (PMC1524979; doi:10.1186/1742-6413-3-14)
Supplement: Additional File 1 — Schematic representation of various cytomorphological patterns observed with ASC-H interpretation. [file 1742-6413-3-14-S1.doc]

| **Figure X:** | | |
| --- | --- | --- |
| PATTERN | **SCHEMATIC REPRESENTATION** | **DESCRIPTION** |
| **REACTIVE**    **ATROPHY** | **Fig-1 Pattern# 1** | **- “Checker board” MGH – like pattern**  - Dark nuclei  - Open chromatin  - Nucleoli.  - Normoblast-like apoptosis confined to the area of nucleus  **- “School of fish” Repair – like pattern**  - Cohesive clusters  - Prominent nucleoli.  - Low N/C ratio  **- Atrophic “ Single-cell” pattern**   - Abundant blue cytoplasm - Open chromatin - W/wout nucleoli   **- Atrophic “ Parabasal cell group” pattern**   - HCG of parabasal cells - Small dark nuclei - Variable, usually scant cytoplasm |
|  | **Fig-2 Pattern# 2** |  |
|  | **Fig -3A Pattern# 3A** |  |
|  | **Fig-3B Pattern# 3B** |  |
| **LSIL pattern** | **Fig-5 Pattern# 5** | **- Cyanophilic Atypical parakeratosis**  - HCG of immature small metaplastic cells  - Sharp angulated cell margins (vs. round peripheral borders of cells at periphery of HSIL syncytium)  - Small koilocytes  - Relatively high N/C ratio  - Chromatin may be smudgy |
| **HSIL pattern** | **Fig-6A Pattern# 6A** | - “**Isolated cell pattern**”   - High N/C ratio - Hyperchromatic nuclei without nucleoli - Coarse chromatin |
|  | **Fig-6B Pattern# 6B** | - **“Syncytial cell” pattern**  - Vague groups of atypical cells  - Focal single-cell apoptosis with random distribution of apoptotic bodies  - Hyperchromatic nuclei without nucleoli  - Coarse chromatin |
